# Redefining distal symmetrical polyneuropathy features in type 1 diabetes: a systematic review

**DOI:** 10.1007/s00592-021-01767-x

**Published:** 2021-07-02

**Authors:** Eleonora Galosi, Xiaoli Hu, Nivatha Michael, Jens Randel Nyengaard, Andrea Truini, Páll Karlsson

**Affiliations:** 1 Department of Human Neuroscience, Sapienza University, Rome, Italy; 2 Core Center for Molecular Morphology, Section for Stereology and Microscopy, Aarhus University, Aarhus, Denmark; 3 Department of Clinical Medicine, Aarhus University, Aarhus, Denmark; 4 Department of Pathology, Aarhus University Hospital, Aarhus, Denmark; 5 Department of Clinical Medicine, Danish Pain Research Center, Aarhus University, Aarhus, Denmark

**Keywords:** Type 1 diabetes, Neuropathy, Distal symmetrical polyneuropathy, Neuropathic pain

## Abstract

Diabetic neuropathy is among the most frequent complications of both type 1 (T1DM) and type 2 diabetes (T2DM) and commonly manifests as a distal symmetrical polyneuropathy (DSPN). Despite evidence that T1DM- and T2DM-related DSPN are separate entities, most of our knowledge on diabetic DSPN derives from studies focused on type 2 diabetes. This systematic review provides an overview of current evidence on DSPN in T1DM, including its epidemiological, pathophysiological and clinical features, along with principal diagnostic tests findings. This review included 182 clinical and preclinical studies. The results indicate that DSPN is a less frequent complication in T1DM compared with T2DM and that distinctive pathophysiological mechanisms underlie T1DM-related DSPN development, with hyperglycemia as a major determinant. T1DM-related DSPN more frequently manifests with non-painful than painful symptoms, with lower neuropathic pain prevalence compared with T2DM-associated DSPN. The overt clinical picture seems characterized by a higher prevalence of large fiber-related clinical signs (e.g., ankle reflexes reduction and vibration hypoesthesia) and to a lesser extent small fiber damage (e.g., thermal or pinprick hypoesthesia). These findings as a whole suggest that large fibers impairment plays a dominant role in the clinical picture of symptomatic T1DM-related DSPN. Nevertheless, small fiber diagnostic testing shows high diagnostic accuracy in detecting early nerve damage and may be an appropriate diagnostic tool for disease monitoring and screening.

## Introduction

Diabetic neuropathy is one of the most common complications of both type 1 (T1DM) and type 2 diabetes mellitus (T2DM), resulting in higher morbidity and mortality, along with massive socio-sanitary burden worldwide [1]. Distal symmetric polyneuropathy (DSPN) is by far the most frequent presentation of diabetic neuropathy [2, 3]. Despite increasing prevalence [4], few studies have specifically addressed type 1 diabetes-related DSPN (T1DM-related DSPN) compared with type 2 diabetes-related DSPN (T2DM-related DSPN), which has been more extensively studied.

There are a number of challenges and unresolved issues regarding diabetic DSPN. Recent evidence questions the pathophysiological role of hyperglycemia in DSPN development. Data from glucose intervention trials have demonstrated glycemic control to be effective in preventing DSPN development in T1DM but not in T2DM, highlighting a possible divergence in T1DM- and T2DM-related DSPN pathophysiological mechanisms [5, 6].

No agreement exists on gold standard testing for DSPN diagnosis. Accordingly, a consensus on DSPN definition has not been reached, though several have been proposed, without any distinction between T1DM- and T2DM-related DSPN [7]. The Toronto consensus criteria are commonly used and require objective diagnostic testing for DSPN diagnosis. In the Toronto criteria, nerve conduction study (NCS) is the gold standard test to assess large fiber damage, whereas intraepidermal nerve fiber density (IENFD) evaluation through skin biopsy and quantitative sensory testing (QST) are the reference methods to assess small nociceptive fibers involvement [8]. A more recent consensus developed by the American Diabetes Association (ADA) suggests a simpler definition of DSPN, as “the presence of symptoms and/or signs of peripheral nerve dysfunction in people with diabetes after the exclusion of other causes,” without electrodiagnostic tests confirmation requirement [9].

Studies have shown that diabetic DSPN commonly displays as a mixed neuropathy, with damage to both sensory large myelinated *Aβ*, small myelinated *Aδ* and non-myelinated C fibers, albeit some patients may suffer from a pure small fiber neuropathy (pure SFN) or a predominantly large fiber one [10, 11]. Patients report of heterogeneous complaints, with negative symptoms like sensory loss and numbness alone or in combination with positive symptoms, such as neuropathic pain and hyperalgesia. Neuropathic pain, traditionally ascribed to small nociceptive fibers damage, is a disabling symptom in many cases [12]. It is still debated to what extent the clinical features of T2DM-related DSPN resemble those of T1DM-related DSPN, which have been less systematically described.

Current knowledge on diabetic DSPN almost completely derives from studies focused on T2DM patients, while few original studies and no systematic reviews have specifically focused on DSPN in T1DM. Given the recent advances highlighting distinctive features of T1DM- and T2DM-associated DSPN [13], herein we systematically review current evidence on DSPN in T1DM, to better define its epidemiological, pathophysiological and clinical features, along with principal diagnostic test findings.

## Methods

Our methodology for the systematic literature search aimed to identify studies on epidemiological, pathophysiological, clinical features, and diagnostic test findings of T1DM-related DSPN, and to exclude studies mainly or exclusively focusing on T2DM. Medline (PubMed), Embase and Cochrane CENTRAL databases were initially searched on March 31, 2020, using the following search strategy: “type 1 diabetes mellitus” as a key word in combination with each of the following in study title or abstract: “neuropathy” OR “polyneuropathy” OR “neuropathic pain.” After the first review of the abstracts, the search was updated on 24 April the same year using the following search strategy: “Diabetic Neuropathies” and “type 1 diabetes” as key words in combination with each of the following in abstract: “neuropathic pain” OR “skin biopsy” OR “laser evoked potentials” OR “corneal confocal microscopy” OR “quantitative sensory testing.” Both clinical and preclinical studies were included and no time limits were set.

Screened abstracts were excluded if they did not focus on T1DM or DSPN. Studies were included if they analyzed patients with T1DM-related DSPN and reported at least one finding pertaining to (1) DSPN epidemiology, (2) DSPN pathophysiology, (3) DSPN clinical manifestations or (4) diagnostic tests findings in DSPN. Studies were excluded if they (1) enrolled fewer than 10 participants per group, (2) reported data exclusively on T2DM patients or did not distinguish between T1DM and T2DM patients in the findings, or (3) were reported as abstracts only. Lastly, we only included studies which relied the DSPN diagnosis on widely accepted diagnostic criteria (e.g., the Toronto consensus criteria, ADA) [8, 9], or objective diagnostic testing (e.g., NCS, QST, skin biopsy), or clinical abnormalities in association with diagnostic testing, or complex combinations of clinical abnormalities, i.e., at least 2 signs and 2 symptoms or elevated scores on the Michigan Neuropathy Screening Instrument (MNSI), the Neuropathy Disability Score (NDS) or the Neuropathy Symptom Score (NSS) [14–16]. Two authors independently assessed the fulfillments of the inclusion criteria.

Data were extracted with a standardized and systematic method, using a spreadsheet with predetermined extraction parameters. The parameters included year of publication, type of study, main topic, number of participants, patient ethnicity, DSPN definition, age, sex, BMI, HbA1c levels, disease duration (diabetes, DSPN and pain, respectively), epidemiology and DSPN risk factors (of painless and painful DSPN, respectively), pain intensity and descriptors, symptoms, signs, neuropathy scores, skin biopsy details (site, methodology and outcomes), corneal confocal microscopy (CCM) results, sensory testing methodology and findings, neurophysiological methodology and findings, as well as any reported associations among the aforementioned parameters. The main findings and outcomes from each study were summarized and presented descriptively.

## Results

**Fig. 1:**
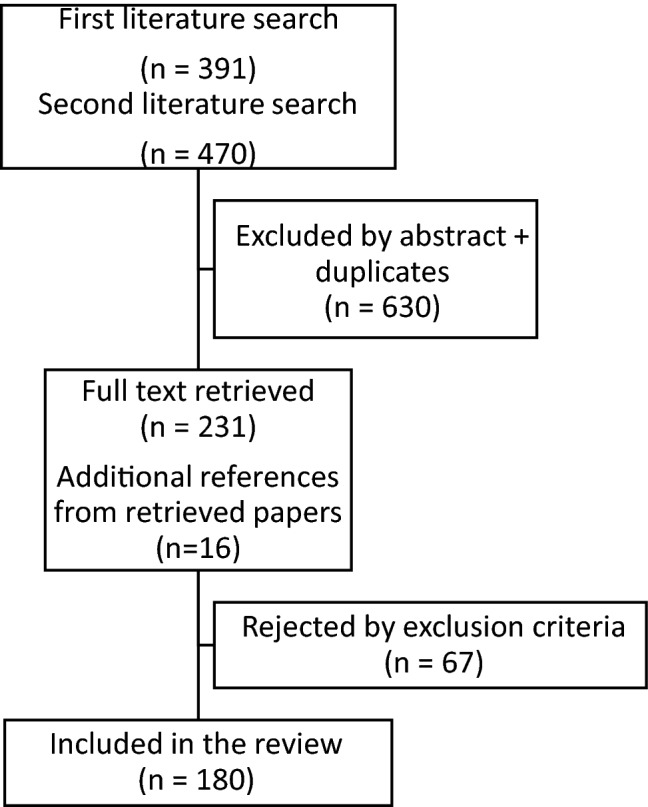
Flowchart of the included and excluded studies in the systematic literature search

Full text was retrieved from 231 out of 861 studies identified in the two literature searches (391 studies in the first search and 470 in the second) and from additional 16 references of retrieved papers. Sixty-seven studies failed to meet the inclusion criteria during data extraction (e.g., data from T1DM patients not reported separately from T2DM), thus resulting in a total of 180 studies included in the review (Fig. 1).

As many as 142 studies (78.9%) were of clinical nature, while the remaining 38 (21.1%) included animal models of T1DM and were categorized as being preclinical. Among the clinical studies, 23 (16.2%) were epidemiological studies, 67 (47.2%) focused on DSPN diagnosis or assessed diagnostic test findings, 40 (28.2%) deepened DSPN pathophysiological mechanisms, and 12 studies (8.4%) regarded other heterogeneous topics. Whereas only three clinical pathophysiological studies focused on painful DSPN, the majority of preclinical studies, as 33 out of 38 (86.8%), focused on the pathophysiology behind neuropathic pain, with only few studies focusing on DSPN pathophysiology. Among clinical studies, 75 interventional studies were identified (52.8%), of which the majority focused on DSPN pathophysiology and diagnosis.

Table 1 summarizes the key symptomatic, functional, and imaging measures used to assess DSPN in the clinical studies included in the review. The most commonly used clinical assessments were ankle reflexes testing (74 studies) and vibration detection threshold measurements (42 studies), while the most commonly used structural parameters were NCS parameters (56 studies), all reflecting function of large myelinated fibers. A wide range of different neuropathy and symptom scoring tools were used. Neuropathy Disability Score (NDS, 29 studies) and Neuropathy Symptom Score (NSS, 16 studies) being the most common ones [16], followed by Michigan Neuropathy Screening Instrument (MNSI, 10 studies) [14]. The most commonly used diagnostic criteria were Toronto consensus criteria (17 studies) [8], followed by American Diabetes Association (4) and San Antonio criteria (3) [9, 17].

**Table 1:** Summary of key symptomatic, functional and imaging measures used to assess and screen for DSPN in the clinical studies included in the review

Neuropathy symptoms and signs scoring (*n* of studies)	Objective functional tests (*n* of studies)	Subjective functional tests (*n* of studies)	Imaging measures (*n* of studies)
NDS (29)	Ankle reflexes (74)	QST (48)	CCM (32)
Toronto consensus criteria (17)	NCS (56)	VDT (42)	Skin biopsy (14)
NSS (16)	Functional MRI (2)	MDT (19)	Nerve biopsy (3)
MNSI (10)	LEPs (1)	Thermal thresholds (14)	Nerve elastography (1)
DN4 (6)	CSP (1)		
ADA criteria (4)			
San Antonio criteria (3)			
LANSS (1)			

*NDS* neuropathy disability score, *NSS* neuropathy symptom score, *MNSI* Michigan Neuropathy Screening Instrument; *DN4* douleur neuropathique en 4 questions questionnaire, *ADA* American Diabetes Association, *LANSS* Leeds assessment of neuropathic symptoms and signs, *NCS* nerve conduction study, *MRI* magnetic resonance imaging, *LEPs* laser evoked potentials, *CSP* cutaneous silent period, *QST* quantitative sensory testing, *VDT* vibration detection threshold, *MDT* mechanical detection threshold, *CCM* corneal confocal microscopy

## Epidemiology

Epidemiological data were retrieved from 23 studies that specifically aimed at analyzing T1DM-related DSPN prevalence and from additional 12 interventional or observational studies reporting DSPN prevalence after consecutive T1DM patients’ enrollment. Out of these 35 studies, 11 focused on pediatric or adolescent patients under the age of 18. Table 2 summarizes the characteristics of the included studies, entailing study type, study size, patient demographics, reported prevalence, and risk factors for DSPN and painful DSPN.

**Table 2:** Prevalence and risk factors for T1DM-related DSPN and painful DSPN in the clinical studies included in the review

Author/Year	Type of study	No. of T1DM patients	Age mean ± SD (years)	Sex (% of males)	T1DM duration mean ± SD (years)	Criteria for T1DM-related DSPN diagnosis	Criteria for painful T1DM-related DSPN diagnosis	DSPN prevalence in T1DM (%)	Painful DSPN prevalence in T1DM (%)	DSPN risk factors in T1DM	Painful DSPN risk factors in T1DM
*Adult patients*											
Adamska et al. (2019)	Interventional, prospective	148	41 (31–49)	58.7	21 (17–30)	Toronto consensus criteria	–	14	–	None	–
Barbosa et al. (2019)	Cross-sectional, retrospective	360	42 ± 14,4	49.4	19.2 ± 12.5	Toronto consensus criteria	DN4 ≥ 4 + LANSS ≥ 12	42.8	18.9% Of T1DM patients	T1DM duration, hypertension	T1DM duration, hypertension, educational level
Falkowski et al. (2019)	Cross-sectional, prospective	100	29 (25–34.5)	53%	12.5 (9–16)	ADA criteria	–	5	–	–	–
Suljic et al. (2019)	Interventional, prospective	30	19.8 ± 3.6	31	4.9	NCS abnormalities	–	23	–	–	–
Andrei et al. (2018)	Interventional, prospective	126	> 18	–	–	ADA criteria	–	25.4	–	T1DM duration, hypertension, age, Hba1c, dyslipidemia, retinopathy, nephropathy	–
Nancy Cardinez et al. (2018)	Cross-sectional, prospective	361	66 ± 9	63	53	MNSI	tingling/pain/ burning/ allodynia in the extremities	42.7	63% of DSPN	-	Female sex
Pan et al. (2018)	Cross-sectional, retrospective	74	–	–	–	ADA criteria	–	21.9	–	T1DM duration, age	–
Truini et al. (2018)	Cross-sectional, prospective	123	47.1 (13.8)	56	23.9 ± 11.8	Toronto consensus criteria	DN4 + clinical examination	23	4.9%		–
Ziegler et al. (2018)	Cross-sectional, prospective	126	59.5 ± 15.4	46.8	–	QST (pressure, temperature, vibration perception)	DSPN with pain and/or burning,	44.3	54.8% of DSPN	–	None
Nybo et al. (2017)	Cross-sectional, prospective	200	55.6	54.3	42	QST (10 g monofilament)	–	53	–	–	–
Pop et al. (2016)	Cross-sectional, prospective	272	44.3 ± 11.9	61.4%	22.7 ± 8	NDS + NSS	–	79.5	–	eGDR, retinopathy, T1DM duration	–
Wang et al. (2016)	Cross-sectional, prospective	314	32.5 ± 9.7	45,8	15.2 ± 9.2	NCS abnormalities	–	22.9	–	Hba1c, hypertension, smoking, cardiovascular disease	–
Bouhassira et al. (2013)	Cross-sectional, retrospective	297	48,3 ± 16	52.2	20.8 ± 12.4	MNSI	DN4 ≥ 4	–	14,7% of T1DM patients	–	–
Araszkiewicz et al. (2011)	Interventional, prospective	140	28	45.7	13 (IQR: 8–19)	Clinical abnormalities + QST (vibration and pressure perception)	–	21.4	–	–	–
Abbott et al. (2011)	Cross-sectional, prospective	1338	37.6 ± 12.9	56.1	17 (10–26)	NDS, NSS, QST (10 g monofilament)	NSS ≥ 5 and NDS ≥ 3	16.2	22.7%	–	Age, female sex, ethnicity
Van Acker et al. (2009)	Cross-sectional, prospective	344	45.9 ± 15	54.2	16.5 ± 17	Neuropen (pain, touch/ pressure perception)	DN4 ≥ 4	25.6	5.8% of T1DM patients	Age, T1DM duration, dyslipidemia, all other diabetes complications	Age, T1DM duration, nephropathy, obesity, dyslipidemia
Beulens et al. (2008)	Cross-sectional, retrospective	1735	39	50	–	Clinical abnormalities + QST (vibration perception)	–	38	–	–	–
González-Clemente et al. (2005)	Cross-sectional, prospective	120	27.17 ± 6.3	54.8	18.9	MNSI	–	30	–	–	–
Tesfaye et al. (2005)	Longitudinal	1172	–	–	–	Clinical abnormalities + QST (vibration perception)	–	28.5	–	Hba1c, T1DM duration. hypertension, smoking, obesity, dyslipidemia, cardiovascular disease	–
Cantòn et al., (2004)	Cross-sectional, retrospective	278	24.8 ± 6.7	56.5	10	San Antonio criteria (NCS, QST, AFT)	–	4.3	–	Hba1c, smoking	–
Shalitin et al. (2002)	Cross-sectional, retrospective	217	23.4 (7,5–49)	47	13.2 (1–34)	NDS	–	17	–	Hba1c, T1DM duration, age	–
Chistyakov et al. (2001)	Cross-sectional, prospective	166	25.0 ± 13.4	58.4	9.6 ± 9.2	Clinical + NCS abnormalities	–	49.4	–	–	–
Christen et al. (1999)	Cross-sectional, retrospective	441	31.4 ± 7.4	75.2	6.5 ± 3.5	Clinical abnormalities (2 symptoms + 2 signs)	–	3.2	–	Hba1c, height, smoking, female sex	–
Young et al. (1993)	Cross-sectional, multicentre study	2426	45(18–90)	53.7	13 (0–62)	Standardized questionnaire and clinical examination	–	22.7	–	Age, T1DM duration	–
*Pediatric patients*											
Sherif et al. (2019)	Cross-sectional, prospective	70	11.9 ± 3.7	34.3%	5.5 ± 3.2	NDS	–	22.9	–	–	–
Ghaemi et al. (2018)	Cross-sectional, prospective	50	16,7 ± 7	46	8.38 ± 3.8	Clinical + NCS abnormalities	DSPN with pain and/or burning	24	25% of DSPN	Hba1c	–
Holiner et al. 2017	Cohort study, prospective	38	12.6 ± 2.4	55.3	5.6 ± 3.2	NCS abnormalities	–	31.6	–	None	–
Türkyilmaz et al. (2017)	Cross-sectional, retrospective	111	11.5	53.2	< 5 (75%)	NCS abnormalities	–	24.3	–	T1DM duration, n of ketoacidosis episodes	–
El-Samahy et al. (2016)	Interventional, prospective	100	14.3 ± 3.3	60	–	NDS + NCS abnormalities	–	23	–	–	–
Louraki et al. (2016)	Cross-sectional, prospective	85	13.5 ± 3.4	52.9	5.5 ± 3.4	NCS abnormalities	–	34.1	–	–	–
Hajas et al. (2016)	Cohort study, prospective	62	13,9 ± 5.9	55	5.6 ± 5.1	Clinical + NCS abnormalities	–	24.2	–	T1DM duration, Hba1c	–
Höliner et al. (2013)	Observational, prospective	39	13.8 ± 2.5	61.5	6.4 ± 3.0	NDS + NSS + NCS abnormalities	–	21	–	–	–
Simsek et al. (2013)	Cross-sectional, retrospective	1032	12.5 ± 4.1	49.5	4.7 ± 3.2	Clinical examination and/or NCS abnormalities	–	1.6	–	T1DM duration, age	–
Jaiswal et al. (2013)	Longitudinal study	329	15.7 ± 4.3	49	6.2 ± 0.9	MNSI	–	8.2	–	Obesity, hypertension, lower HDL [1]	–
Ferreira et al. (2005)	Cross-sectional, prospective	48	12.9 ± 3.5	58	7 ± 2.5	Dick’s criteria (NCS and clinical examination)	–	25	–	–	–

*T1DM* type 1 diabetes mellitus, *NCS* nerve conduction study, *QST* quantitative sensory testing, *AFT* autonomic function tests, *NDS* Neuropathy Disability Score, *NSS* Neuropathy Symptom Score, *DN4* douleur neuropathique en 4 questions questionnaire, *LANSS* Leeds assessment of neuropathic symptoms and signs, *MNSI* Michigan Neuropathy Screening Instrument, *ADA* American diabetes association, *eGDR* estimated glucose disposal rate (insulin resistance)

A high heterogeneity was observed in T1DM-related DSPN prevalence in pediatric patients, ranging between 1.6 and 34.1%, although eight of the 11 pediatric studies (73%) used homogeneous diagnostic criteria (NCS abnormalities). We performed a meta-analysis to estimate the prevalence of T1DM-related DSPN in pediatric patients, including the eight studies that used NCS to diagnose DSPN. The prevalence among the eight studies was estimated to be 25.9% (range 21–34.1%). The studies included a total of 533 T1DM pediatric patients, of which 138 were diagnosed with DSPN.

Studies assessing T1DM-related DSPN prevalence in adults (*n *= 24) were more heterogeneous in selection criteria, making comparisons and prevalence assumptions difficult.

Only two cross-sectional prospective studies (8.7%) used Toronto consensus criteria, reporting a prevalence of 14% and 23%, respectively [18, 19]. Three studies (12.5%), including between 74 and 126 patients, used ADA criteria without any diagnostic test confirmation and reported a prevalence between 5 and 25.4%[20–22]. Higher prevalence was found in six adult studies using a combination of clinical and QST abnormalities for DSPN diagnosis (21.4–44.3%) and in two studies relying only on MNSI (30–42.7%) [23, 24]. Out of five studies reporting a prevalence higher than 40%, four studies included patients with long diabetes duration (over 20 years) [25–28].

Only one epidemiological study assessed pure small fiber neuropathy prevalence in T1DM, reporting that it was present in 0% of the study population, meaning that all of the 28 included patients with T1DM-related DSPN had large fiber involvement [19].

Seven epidemiological studies in adult patients compared the prevalence between T1DM- and T2DM-related DSPN and all found that DSPN frequency was significantly higher T2DM compared with T1DM [19–21, 25, 29–31]. DSPN prevalence in T2DM exceeded that in T1DM by 10–25% throughout these studies, with two studies finding that DSPN prevalence in T2DM almost doubled the prevalence of DSPN in T1DM (25.6 vs. 50.8%, 28.7 vs. 50.7%, respectively) [20, 29]. By using multivariate analysis, four of the studies confirmed that diabetes type was independently associated with DSPN after correction for age, diabetes duration and other factors [20, 29–31]. Diabetes duration was the most frequently reported risk factor for T1DM-related DSPN (11 studies), followed by HbA1c (8 studies), age (6 studies), hypertension (5 studies), smoking (4 studies), retinopathy (3 studies), nephropathy (3 studies) and dyslipidemia (3 studies). No significant differences in risk factors emerged between children and adult patients, but diabetes duration and Hba1c level were the most frequently reported in all age groups.

## Pathophysiology

A total of 40 clinical and 39 preclinical studies focused on DSPN pathophysiology in T1DM. While 37 clinical studies (92.5%) focused on DSPN pathophysiology itself and only three (7.5%) on neuropathic pain, 33 (85%) of preclinical studies focused on neuropathic pain pathogenesis. In general, each study focused on a specific possible pathophysiological mechanism, thus resulting in somewhat fragmented evidence. Below we highlight findings from two main topics: hyperglycemia and inflammation.

Some studies focused on hyperglycemia’s role as a major determinant for DSPN in T1DM [32], by demonstrating that prolonged hyperglycemia may lead to nerve damage by raising reactive oxygen species (ROS) concentration and oxidative stress [33, 34], and enhancing the production of advanced glycation end products [35, 36]. Other studies focused on the role of chronic inflammation leading to DSPN [37], some of them underlining the importance of activation of the TNF-alpha system [24, 38] and the enhanced expression of pro-inflammatory cells, possibly influenced by persistent poor glycemic control [39]. Endothelial dysfunction, caused by chronic inflammation, has also been identified as a cornerstone in the development of diabetic complications, including DSPN [18, 40, 41]. One study hypothesized that autoantibodies against glutamic acid decarboxylase and islet antigen-2 are involved in the development of axonal degeneration in T1DM-related DSPN, possibly representing a peculiar pathophysiological mechanism in T1DM [42].

Regarding painful DSPN, most animal studies noted the relation between neuropathic pain and oxidative stress [43–46], which was consistently reported to be associated with inflammatory factors release, i.e., TNF-alpha and IL1-beta production after activation of p38-MAPK and PKC pathways [38]. One study suggested that sorbitol accumulation in nerve cells contributes to neural damage and neuropathic pain development, by increasing cellular osmolarity and inflammatory injury [47].

## Clinical features

### Painful and non-painful symptoms

Out of 142 clinical studies, few reported the prevalence of painful and non-painful symptoms in T1DM-related DSPN. Twelve studies reported neuropathic pain prevalence in T1DM-related DSPN, ranging between 0 and 54.8%. However, only three studies, all assessing adult patients, used the DN4 questionnaire for neuropathic pain identification in patients with DSPN, reporting a prevalence of painful DSPN between 5.8 and 18.9% [26, 29, 48]. Four studies in total, using widely agreed criteria for neuropathic pain diagnosis (questionnaires + clinical examination), compared neuropathic pain prevalence in T1DM- and T2DM-related DSPN and found lower neuropathic pain prevalence in T1DM compared with T2DM-related DSPN [25, 29, 48, 49]. One of these studies [48] reported neuropathic pain subtypes frequency and severity as assessed by DN4, and found that most patients with painful DSPN suffered from pain of moderate to severe intensity, with ongoing burning pain being the most common type.

Four studies reported risk factors for neuropathic pain in T1DM-related DSPN. The most common ones were diabetes duration (2 studies), age (2 studies), and female sex (2 studies).

Among non-painful symptoms, numbness was the most frequently mentioned symptom (9 studies), with a reported frequency in T1DM-DSPN ranging between 13.3 and 65.7%. Out of five studies reporting the frequency of both numbness and neuropathic pain in T1DM, four reported numbness being more common compared with neuropathic pain in T1DM-DSPN [25, 50–52].

Several studies assessing diagnostic tests findings in children with T1DM showed a high prevalence (up to 87%) of subclinical asymptomatic T1DM-DSPN, as diagnosed with different instrumental tests [42, 53–58]. Similarly, two studies in adult patients showed high prevalence rates of subclinical DSPN (53–96.6%) as diagnosed by NCS [57, 59].

### Objective clinical signs

Though ankle reflexes assessment was the most commonly used bedside clinical examination test to assess and screen for T1DM-related DSPN, only 9 studies reported the frequency of ankle reflexes absence or reduction, ranging between 2 and 75% in patients with diabetic DSPN. The two studies reporting the lowest prevalence of ankle reflexes abnormalities (2–15%) included only pediatric patients [60, 61], but other studies on pediatric patients found abnormalities in high percentages of patients (65–75%) [50, 62].

Vibration hypoesthesia and vibration detection threshold were frequently assessed by clinical examination in our selected studies (42 studies). However, only 14 studies reported vibration hypoesthesia frequency, ranging between 5.1 and 69%, with heterogeneous tools used to detect vibration sensitivity. Only three studies used the 128 Hz tuning fork, reporting vibration hypoesthesia in 24.8–41.7% of the patients with diabetic DSPN. Five studies used a biothesiometer, while six used different vibrometers, finding highly variable prevalence of abnormalities.

The frequency of thermal sensitivity abnormalities, as assessed with different methods, was reported by eight studies, with thermal hypoesthesia presence ranging between 8.3 and 43.8%. Four studies assessed warm and cold detection thresholds using thermal probes, reporting thermal hypoesthesia being present in 12.5–27.5% of T1DM patients, except for one study including patients with longer diabetes duration (over 20 years), which reported higher prevalence (41%). Other four studies, assessing the ability to discriminate warm and cold sensation through a Tip-Therm device [54] or by comparing warm and cold water [50], found lower prevalence of thermal sensitivity abnormalities (8.3–12%), apart for one study reporting high frequency (43.8%) in patients with higher age and long diabetes duration [21].

Three studies assessed pinprick hypoesthesia, hereof two studies that reported pain hypoesthesia in 0–15% of patients and one study, including the patients with the longest diabetes duration (over 20 years), reported pinprick sensation impairment in 23% of the patients.

Only one study systematically compared the frequency of clinical signs between patients with T1DM- and T2DM-related DSPN and reported slightly higher frequency of ankle reflexes reduction, vibration, thermal and pinprick hypoesthesia in T2DM-related DSPN [21]. Few studies reported motor signs prevalence, which homogeneously resulted to be below 5%.

## Diagnosis

Diagnostic criteria for DSPN varied greatly among the studies, ranging from one single test (e.g., ankle reflexes, vibration threshold, or NCS) to a definite diagnosis following the Toronto consensus criteria. Table 3 summarizes the most frequently used criteria for DSPN diagnosis in the clinical studies included in the review. Out of 142 clinical studies, at least 37 (26.1%) used one single test or questionnaire to diagnose DSPN. Seventeen studies diagnosed DSPN based on a single questionnaire, such as MNSI, NDS, or NSS, which, in fact, were not developed as diagnostic tools but as screening tools [51, 63]. Some clinical studies are based on DSPN diagnosis on single items of QST, where the most frequently “diagnostic” tests were vibration threshold (5 studies) and tactile threshold (2 studies), reflecting large myelinated fibers function. The most commonly used objective test to diagnose DSPN in clinical studies was NCS (*n* = 33), assessing large myelinated motor and sensory fibers. Very few studies included small fiber-related objective testing in the diagnostic criteria, like QST thermal thresholds or skin biopsy, which is the gold standard neuropathological test for small fibers. Eighteen studies used Toronto consensus criteria as diagnostic criteria for patient enrollment; however, only few of these studies were based on a definite DSPN diagnosis according to Toronto criteria, requiring skin biopsy and/or QST.

**Table 3:** Criteria used for T1DM-related DSPN diagnosis in the clinical studies included in the review

Diagnostic criteria	Total clinical studies, *n* (%)	Epidemiological studies, *n*	Pathophysiological studies, *n*	Diagnostic tests studies, *n*	Other studies, *n*
Neuropathy symptoms and/or signs	20 (14.08%)	2	6	9	3
Toronto consensus criteria	18 (12.68%)	2	4	10	2
NCS + neuropathy symptoms and/or signs and/or questionnaires (NDS/NSS)	16 (11.26%)	2	4	7	3
NCS	13 (9.15%)	3	4	5	1
NDS	9 (6.33%)	1	5	3	0
QST, 1 test	8 (5.63%)	0	6	1	1
MNSI	6 (4.22%)	3	1	0	2
ADA	4 (2.82%)	2	2	0	0
NDS + NSS	4 (2.82%)	1	1	2	0
NCS + QST + neuropathy signs or questionnaires	4 (2.82%)	0	0	4	0
San Antonio criteria	3 (2.11%)	2	1	0	0
NSS	2 (1.41%)	0	0	2	0
QST, 2 tests	2 (1.41%)	1	0	1	0
QST, 3 tests	2 (1.41%)	1	1	0	0
QST + neurological examination abnormalities and/or questionnaires	2 (1.41%)	1	0	1	0
Other criteria	29 (20.42%)	2	5	22	0
Clinical studies for each category, *n*	142 (100%)	23	40	67	12

*NCS* nerve conduction study, *NDS* Neuropathy Disability Score, *NSS* neuropathy symptom score, *QST* quantitative sensory testing, *MNSI* Michigan Neuropathy Screening Instrument, *ADA* American Diabetes Association

### Nerve conduction studies

NCS was the most commonly used test to screen for and to diagnose DSPN. However, among 56 clinical studies performing NCS (39.4%), only 24 studies described NCS findings in detail, allowing a better neurophysiological characterization of T1DM-related DSPN. Sensory and motor nerve conduction velocities were the most frequently used outcome (13 studies), followed by action potentials amplitudes (10 studies).

Few studies described the neurophysiological profile of a predominantly sensory axonal polyneuropathy [64–66]. Three studies revealed that NCS abnormalities in motor nerves were more frequent than changes in sensory nerves [60, 67, 68]. Two of these studies also analyzed T2DM patients and showed greater impairment of motor nerve parameters in T1DM compared with T2DM, without significant differences in sensory parameters. Two additional studies performing motor unit estimation (MUNE) assessed an early motor unit loss in T1DM-related DSPN, even at preclinical stages (i.e., asymptomatic children with short diabetes duration)[69, 70]. Many studies revealed NCS abnormalities also in asymptomatic patients, indicating that NCS may be an essential tool in detection of subclinical DSPN. Furthermore, two longitudinal studies evaluating the time course of NCS changes in T1DM patients showed that NCS abnormalities incidence doubled in 5–10 years, mostly due to subclinical stages with Hba1c being the most frequently reported factor associated with conduction parameters deterioration [53, 54].

### Skin biopsies

A total of 17 studies performed skin biopsy analysis, of which three were preclinical. However, only 11 studies followed the European Federation of the Neurological Societies (EFNS) guidelines for skin biopsy treatment and determination of intraepidermal nerve fiber density (IENFD) [71]. The remaining six studies performed alternative analyses other than immunohistochemical staining of intraepidermal nerve fibers (e.g., Western blot analysis or measurement of epidermal thickness).

All eight clinical studies that compared IENFD between study participants with and without T1DM found a significant IENFD decrease in patients. Only three studies examined differences between T1DM patients with and without DSPN, and found lower IENFD in patients with DSPN compared to those without. No study assessed possible IENFD differences between painful and painless DSPN. In general, the studies reported a uniform loss of fibers, worsening along with neuropathy severity.

In contrast to T2DM, very few additional analyses other than IENFD evaluation have been performed on skin biopsies from patients with T1DM, such as axonal swellings assessment or additional staining for pain or autonomic markers, with only one study looking at GAP-43 [72]. A single study analyzed IENFD differences between T1DM and T2DM diabetes patients, reporting no difference [65]. One study used topical capsaicin for nerve fiber depletion and tested regeneration rate by taking a baseline biopsy prior to the treatment and again 12 months later [73]. The study showed that the IENFD in the follow-up biopsy was over 90% compared with the baseline biopsy in non-diabetic controls, but only 76% for patients with T1DM and as low as 58% for patients with T2DM. Duration of diabetes was associated with slower regeneration rate in the T1DM patients. Similarly, a 5-year follow-up study [74] found in both T1DM and T2DM diabetes patients a steady IENFD decline, faster than one would expect in non-diabetics, albeit faster in T2DM. Lastly, a study reported that a simultaneous pancreas and kidney transplantation was associated with early and maintained small nerve fiber regeneration in the cornea and the skin [72].

### Corneal confocal microscopy (CCM)

A total of 33 studies performed CCM, of which 32 were clinical studies, hereof two in adolescents. However, only 12 studies compared CCM measures in patients with and without DSPN, and no study assessed possible differences between painful and painless DSPN.

Measures from CCM can either be determined manually or by a software allowing automatic quantification. However, normative reference values are available only for manual CCM. One study set upper (> 15.8) and lower (< 11.8) corneal nerve fiber length (CNFL) thresholds for diagnosis of DSPN in type 1 diabetes, with 91% sensitivity and 93% specificity [75]. Thresholds for diagnosis are not available for corneal nerve fiber density (CNFD) or corneal nerve fiber branch density (CNBD). Ten out of 14 clinical studies (72%) assessing CNFD reported a decreased CNFD in patients with T1DM compared with non-diabetic controls. The same was the case for 11 out of 13 studies assessing CNFL (84.6%), and eight out of 12 (66.7%) for studies assessing CNBD.

Six out of seven studies that directly compared CCM measures between T1DM patients with and without DSPN found a difference [75–80]. The study failing to find any difference between patients with and without DSPN, was performed in adolescents with shorter DSPN duration [81]. Not all studies measured the traditional CCM measures (NCFD, CNFL, or CNBD), but used instead other types of measures, including “number of long nerve bundles” and sub-basal nerve (SBN) density.

A study testing the efficacy of omega-3 supplementation on DSPN used CCM as a primary outcome after intervention, reported that CNFL and CNBD increased at 12 months compared with baseline [82]. Two studies used CCM together with skin biopsy to detect small fibers regeneration after simultaneous pancreas and kidney transplantation [72, 83]. Six studies performed both CCM and skin biopsies for IENFD quantification [64, 72, 77–79, 83]. The studies found comparable diagnostic effectiveness between CCM and IENFD. Specifically, one study reported that CNFD had a sensitivity of 0.77 and a specificity of 0.79 for DSPN diagnosis, with an area under the ROC curve of 0.81. In turn, IENFD showed a sensitivity of 0.61 and a specificity of 0.80, with an area under the ROC curve of 0.73 [79].

### Quantitative sensory testing (QST)

A total of 48 clinical studies (33.8%) performed QST and reported findings. However, all except two studies performed very limited sensory testing. Only one study performed the full, 13-item long QST protocol issued by the German research network on neuropathic pain [84] and additional one study performed the majority of the protocol [85]. Most studies performed only one or two tests, typically vibration and mechanical detection threshold (VDT and MDT). Results were not always included, e.g., in studies where QST was used to determine or screen for DSPN presence. When results were presented, differences emerged in QST parameters between patients with and without DSPN, indicating sensory dysfunction in both large and small nerve fibers [84, 86–88]. Both small and large fibers were affected in T1DM-related DSPN patients, even in young patients showing no symptoms [89], showing twice the prevalence of QST abnormalities compared with clinical examination [86]. Thermal thresholds were abnormal in up to 86% of DSPN patients in one study [87]. QST was also the most frequently used method to show DSPN presence in animal models of neuropathy, appearing in additional 20 preclinical studies. Most frequently used sensory methods in animals were von Frey hairs, hot plate and Hargreaves.

## Discussion

While there have been recently published important reviews in the field of diabetic DSPN with different aims, including disease mechanisms [90], management [9], screening strategies [91–93], biomarkers and diagnostic tools [92, 93], and pharmacological treatment of neuropathic pain [94], none have focused on DSPN in T1DM. Most of our knowledge on diabetic DSPN derives from studies focusing on T2DM patients, even though T1DM and T2DM are recognized as two distinctive entities [95].

This systematic review aimed to provide an overview of current evidence on DSPN in T1DM, including its epidemiological, pathophysiological and clinical features, along with principal diagnostic test findings. As shown in Table 4, the main findings were that DSPN seems to be less prevalent in T1DM compared with T2DM, and there also appears to be some differences in risk factors of developing DSPN between T1DM and T2DM. Just as in T2DM, diabetes duration, age, and HbA1c are the most frequently reported risk factor for T1DM-associated DSPN, while BMI and male sex, two frequently reported risk factor for T2DM-associated DSPN, were never mentioned as risk factors for developing DSPN in T1DM. There is some evidence that painful DSPN is less common in T1DM compared with T2DM. The overt clinical picture of T1DM-related DSPN seems to be dominated by large fiber-related clinical signs and non-painful symptoms, with many cases having an asymptomatic course.

**Table 4:** Summary of key epidemiological and clinical findings regarding T1DM-related DSPN

Analyzed variables in T1DM-related DSPN	% (*n* of studies)	Other observations (*n* of studies)
DSPN prevalence in adults	14–44.3% (19)	Lower prevalence of DSPN in T1DM compared with T2DM (7)
DSPN prevalence in childhood	25.9% [21–34.1%] (8)*	T1DM-related DSPN prevalence in childhood is slightly lower than in adults
Neuropathic pain prevalence	5.8–18.9% (3)**	Neuropathic pain prevalence is lower in T1DM compared with T2DM-related DSPN (4)
Non-painful symptoms prevalence	13.3–65.7% (9)	Non-painful symptoms are more frequent than neuropathic pain in T1DM-related DSPN (4)
Subclinical neuropathy prevalence	35–96.6% (8)	-
Ankle reflexes abnormalities frequency	2–75% (9)	Frequency of large fiber-mediated signs (ankle reflexes abnormalities and vibration hypoesthesia) is higher compared with small fiber-related signs (thermal and pinprick hypoesthesia) in T1DM-related DSPN
Vibration hypoesthesia	5.1–69% (14)	
Thermal hypoesthesia	8.3–43.8% (8)	
Pinprick hypoesthesia	0–23% (3)	
Main risk factors for DSPN	T1DM duration (11), HbA1c (8), age (6), hypertension (5), smoking (4), retinopathy (3), nephropathy (3), dyslipidemia (3)	BMI and male sex, two frequently reported risk factors for T2DM-related DSPN, were never reported for T1DM-related DSPN
Main risk factors for neuropathic pain	Diabetes duration (2), age (2), female sex (2)	T1DM- and T2DM-related DSPN share risk factors for neuropathic pain

(*n* studies) is the number of studies reporting the analyzed variable. *DSPN prevalence in childhood was calculated through a meta-analysis of 8 studies based on similar diagnostic criteria; **Neuropathic pain prevalence estimation was based on the 3 studies using the DN4 questionnaire for neuropathic pain diagnosis, a widely agreed screening tool for neuropathic pain.

The sections below discuss the findings in this review in detail.

## Epidemiology and risk factors

There was a high degree of heterogeneity in estimations of prevalence of T1DM-related DSPN in adults, likely due to different selection criteria between studies. However, the most accurate studies, using a combination of clinical and instrumental abnormalities for DSPN diagnosis, reported a prevalence range of 14–44.3%, with two cross-sectional prospective studies [18, 19] using Toronto consensus criteria estimating a prevalence of 14% and 23%.

The 7 studies in adult patients that compared DSPN prevalence between T1DM and T2DM, all found DSPN to be less common in T1DM, as previously demonstrated by another systematic review [96]. As hypothesized in some studies [20, 31], a possible explication for this prevalence difference could reside in pathophysiological mechanisms, with other factors beyond hyperglycemia possibly implicated in the development of DSPN in T2DM, such as metabolic syndrome and its components. Given that diabetes duration is a risk factor for both T1DM- and T2DM-related DSPN, the higher prevalence of T2DM-related DSPN could also be due to a longer subclinical disease duration in T2DM, with longer prodromal periods between the onset and the diagnosis of diabetes.

There was also a high degree of heterogeneity in prevalence of DSPN in the pediatric and adolescent population. However, a meta-analysis including 8 studies based on similar diagnostic criteria found a prevalence of 25.9%, suggesting a slightly lower prevalence in pediatric patients compared with adults [49], which is likely explained by shorter diabetes duration in the younger population. The second-most frequently reported risk factor after diabetes duration was HbA1c, corroborating the common belief that hyperglycemic damage plays a pivotal role in T1DM-related DSPN pathogenesis [90]. Dyslipidemia was rarely reported as a risk factor, probably due to young patients enrollment in most studies, while BMI and male sex, two frequently reported T2DM-related DSPN risk factors [97], were never reported for T1DM-related DSPN in the included studies. These findings suggest that metabolic syndrome factors, commonly associated with T2DM-DSPN, play a negligible role in T1DM-related DSPN development.

## Pathophysiology

Although DSPN pathogenesis is not fully understood, different pathophysiological mechanisms have been hypothesized in T1DM- and T2DM-related DSPN [13]. Hyperglycemia has traditionally been considered a major determinant for diabetic neuropathy; however, its contribution is probably higher in T1DM, given that recent meta-analysis have demonstrated that tight glucose control can improve polyneuropathy in T1DM, showing no efficacy in T2DM [5, 6]. Among the included studies in this review, hyperglycemia is a largely recognized risk factor for T1DM-DSPN.

Prolonged hyperglycemia may lead to nerve damage by raising reactive oxygen species (ROS) and oxidative stress through increased glycolysis activation [33, 34] and by enhancing the production of advanced glycation end products (AGEs), with protein and cellular dysfunction as a consequence [35, 36]. Furthermore, raised flux through the polyol and the hexosamine pathway contribute to neural damage, by increasing cellular osmolarity and causing inflammatory injury [90].

Several studies found an association between T1DM-related DSPN and hypertension [20, 26, 30, 98, 99], thus suggesting that hypertension-induced microvascular damage could contribute to nerve injury in T1DM-related DSPN. It is still unclear whether hypertension acts a risk factor in T2DM-related DSPN, given that the association has been less consistently reported in T2DM [100–102].

Differences in insulin signaling pathways may also account for diversity in T1DM- and T2DM-related DSPN pathophysiology. Reduction in neurotrophic insulin effects, due to insulin deficiency in T1DM or to insulin resistance in T2DM, are thought to contribute to diabetic neuropathy pathogenesis [90, 103]. Furthermore, autoimmune mechanisms are hypothesized to contribute to neural damage in T1DM, as the presence of autoantibodies against glutamic acid decarboxylase (GAD) and islet antigen-2 (IA-2) could represent a distinct pathophysiological mechanism for axonal degeneration development in T1DM-associated DSPN [42]. C-peptide reduction may be an additional contributor to nerve dysfunction peculiar to T1DM, via reduction in Na/K ATPase activity and endothelial nitric oxide synthase (eNOS) activity, with resulting hindrance to endoneurial blood flow [104].

All these mechanisms, leading to mitochondrial dysfunction and DNA damage with recruitment of inflammatory mediators and cellular death, converge in the clinical picture of diabetic neuropathy [103], affecting not only neurons, but also glial cells and endothelial ones, with microcirculatory dysfunction [18, 40]. Even Schwann cells are affected and demyelinating features may be present in most severe cases, despite diabetic neuropathy being considered a primarily axonal neuropathy [103].

Unlike T2DM-related DSPN, pathophysiology of neuropathic pain in T1DM-related DSPN has not been adequately explored in human studies, remaining largely unexplained why some patients develop neuropathic pain and others do not. Complex coaction of genetic, psychological and metabolic factors, and neuropathy severity, has been suggested to play a critical role [105].

The majority of preclinical studies on painful DSPN in T1DM highlighted the relationship between neuropathic pain and oxidative stress [43–46], consistently associated with inflammatory factors release (i.e., TNF-alpha and IL1-beta production after p38-MAPK and PKC pathway activation) [38]. Several studies also showed that sorbitol accumulation in nerve cells is closely related to neuropathic pain development, accompanied by endothelial alterations and a decline in microvascular integrity at the dorsal horn and terminal axon [106].

## Clinical features

The few studies reporting neuropathic pain prevalence in T1DM-related DSPN, by using widely accepted criteria for neuropathic pain identification, reported a prevalence of painful DSPN between 5.8 and 18.9% [26, 29, 48]; four out of five studies comparing neuropathic pain prevalence in T1DM- and T2DM-related DSPN, found lower neuropathic pain prevalence in T1DM [25, 29, 48, 49]. The few studies analyzing risk factors for neuropathic pain reported painful T1DM-related DSPN being associated with increased disease duration, female sex, dyslipidemia, and other diabetes complications such as nephropathy, not differently from T2DM-related DSPN [19, 29]. The relation between neuropathic pain and diabetes duration, dyslipidemia, and diabetic complications, which are usually higher in T2DM patients, could partially account for higher neuropathic pain prevalence in T2DM-related DSPN. Furthermore, the association between pain and diabetes duration, together with dyslipidemia and nephropathy, two comorbidities that are more likely present in advanced cases, suggest that neuropathic pain characterizes more progressed and severe cases of diabetic neuropathy, as already shown for T2DM-related DSPN [49, 107, 108].

The majority of the included studies demonstrated non-painful symptoms, like numbness, being more common than neuropathic pain in T1DM-associated DSPN.

Several studies, especially in young patients, reported very high frequencies of subclinical neuropathy, thus suggesting that many cases of T1DM-related DSPN might go unnoticed due to lack of clinical disturbances and signs [42, 53–58].

Regarding clinical examination findings, higher frequencies were reported for objective signs of large fibers dysfunction, such as reduction in ankle reflexes and vibration hypoesthesia, compared with signs of small fiber damage (e.g., thermal and pain hypoesthesia). However, signs of small fiber impairment were assessed by only few studies and with inhomogeneous methods.

These findings as a whole suggest that T1DM-related DSPN is a frequent and often subclinical T1DM complication, with an overt clinical picture less frequently characterized by neuropathic pain respect to T2DM-related DSPN. The lower neuropathic pain prevalence in T1DM-related DSPN, the higher frequency of non-painful compared with painful symptoms, and the higher prevalence of large fiber-related clinical signs in comparison with small fibers ones, all indicate that large fiber impairment could play a dominant role in the clinical picture of symptomatic T1DM-related DSPN.

## Diagnostic test findings

Diagnostic tests and inclusion criteria currently used in clinical studies vary greatly [109] [7] [71, 110], resulting in heterogeneous definition and varying prevalence of T1DM-related DSPN. The majority of the studies used a combination of clinical abnormalities and diagnostic testing to diagnose T1DM-related DSPN, with NCS, assessing large myelinated nerve fibers, the most frequently used method. Small fiber damage was rarely considered in the studies’ inclusion criteria.

It has, for a long time, been speculated if small or large fiber damage happens first in DSPN and the findings have been contradictory. In the vast majority of studies, NCS (measuring large fibers function), skin biopsy and CCM (measuring small fibers damage), and QST (evaluating both fiber types), showed relevant abnormalities not only in patients suffering from T1DM-related DSPN, but also in asymptomatic patients with T1DM [56, 58, 59]. However, two studies provided evidence that small fiber impairment precedes large fiber one [111, 112], suggesting that small fiber investigations, such as skin biopsy, QST, and CCM, could be appropriately used for screening of patients.

Detailed NCS studies described T1DM-related DSPN as a predominantly length-dependent sensory axonal polyneuropathy, similar to T2DM-related DSPN [64–66]. Interestingly, several studies showed more prominent NCS abnormalities in motor nerves compared with sensory nerves [60, 67, 68], and early motor unit loss as assessed by MUNE being present at preclinical stages of T1DM-related DSPN [69, 70]. Furthermore, two studies showed greater impairment of motor nerve parameters in T1DM-related DSPN compared with T2DM-related DSPN, without significant differences in sensory parameters [67, 68].

The topic if motor seems to prevail on sensory nerve involvement is highly debatable. The aforementioned findings, suggesting early and more pronounced motor impairment in T1DM-DSPN, are far from demonstrating that motor involvement is a crucial feature in T1DM-related DSPN, since neurophysiological motor parameters abnormalities are not associated with relevant clinical motor impairment. However, they highlight the relevance of large myelinated fiber damage in T1DM-related DSPN, also supported by clinical and histopathological studies. One study, after detailed QST and NCS assessment, demonstrated a more accentuated loss of myelinated nerve fibers in T1DM patients [65] and a more severe reduction in unmyelinated nerve fibers in T2DM [74]; another study, separately comparing mechanoreceptive Meissner corpuscle innervation and IENFD in T1DM and T2DM cohorts, revealed a more severe loss of myelinated nerve fibers in T1DM patients [68]. These findings, together with the higher prevalence of large fiber-mediated signs, such as reduction in ankle reflexes and vibration hypoesthesia, along with the greater frequency of non-painful over painful complaints, suggest that large fiber damage plays a prominent role in T1DM-DSPN characterization. However, other studies contemporarily assessing both small and large fibers function with diverse methods, reported higher prevalence of small fiber abnormalities compared with large fibers ones [113–115], thus making assumptions on neural damage distribution difficult. Further studies, using recommended diagnostic tools for large and small fiber assessment, are needed to better understand which fiber types are affected most.

Skin biopsies and CCM are commonly used methods to assess morphological damage of small fibers in neuropathies. Both methods have been shown to have comparably high diagnostic effectiveness [78, 79]. Specifically, one study reported that CNFD had a 0.77 sensitivity and a 0.79 specificity for DSPN diagnosis, while IENFD showed a sensitivity of 0.61 and a specificity of 0.80 [79]. Larger studies are though needed to truly reveal their clinical usefulness in T1DM-associated DSPN. One study compared IENFD between T1DM and T2DM, finding no significant differences [65], while two studies assessing nerve fiber regeneration rate in diabetes found a stronger impairment in T2DM compared with T1DM, possibly suggesting a more severe small fiber damage in T2DM [73, 74].

CCM has been used to monitor nerve regeneration in several studies and clinical trials [72, 82, 83]. Due to its less invasiveness and comparable diagnostic accuracy respect to skin biopsy, CCM can be considered as an appropriate tool for disease monitoring and screening, especially in pediatric patients, as well as a valid outcome measure in clinical trials.

## Limitations in knowledge

Our systematic revision of epidemiological data was troubled with lack of uniform definitions and diagnostic criteria for diabetic DSPN and painful DSPN, resulting in heterogeneous results, with broad prevalence ranges both for T1DM-related DSPN and for neuropathic pain.

Heterogeneous diagnostic testing was used in the analyzed studies, with relatively few studies using widely recommended diagnostic tests, particularly concerning small fiber damage assessment, which needs to be implemented by further studies.

Furthermore, most studies are relatively small and of cross-sectional design, with a lack of clinical studies addressing pain pathophysiology in T1DM-associated DSPN, as we only identified three studies on this topic. On a similar note, little is known about risk factors of developing painful DSPN in T1DM, as only five studies addressed risk factors of painful DSPN.

## Conclusions

This systematic review of T1DM-related DSPN shows that DSPN is a less frequent complication in T1DM compared with T2DM, and that both types share common risk factors for development of DSPN. Even so, there are distinctive pathophysiological mechanisms that underlie T1DM-related DSPN development, with hyperglycemia being a major determinant. Reduction in neurotrophic insulin effects, C-peptide deficiency, and autoimmune-mediated neural damage could represent specific pathogenetic mechanisms for T1DM-related DSPN, whereas it remains largely unexplained why some patients suffer from neuropathic pain. The lower neuropathic pain prevalence in T1DM-related DSPN compared with T2DM-associated DSPN, the higher frequency of non-painful compared with painful symptoms, and the higher prevalence of large fiber-related clinical signs in comparison with small fibers ones, all indicate that large fiber impairment could play a dominant role in the clinical picture of symptomatic T1DM-related DSPN. However, small fiber damage assessment shows high diagnostic accuracy in detecting early damage and may be an appropriate diagnostic tool for disease monitoring and screening.
